# The home environment and childhood obesity in low-income households: indirect effects via sleep duration and screen time

**DOI:** 10.1186/1471-2458-14-1160

**Published:** 2014-11-09

**Authors:** Bradley M Appelhans, Stephanie L Fitzpatrick, Hong Li, Vernon Cail, Molly E Waring, Kristin L Schneider, Matthew C Whited, Andrew M Busch, Sherry L Pagoto

**Affiliations:** Department of Preventive Medicine, Rush University Medical Center, 1700 W. Van Buren St., Suite 470, Chicago, IL 60612 USA; Department of Quantitative Health Sciences, University of Massachusetts Medical School, Worcester, MA USA; Department of Psychology, Rosalind Franklin University, North Chicago, IL USA; Department of Psychology, East Carolina University, Greenville, NC USA; Department of Psychiatry and Human Behavior, Brown University, Providence, RI USA; The Miriam Hospital, Providence, RI USA; Division of Preventive and Behavioral Medicine in the Department of Medicine, University of Massachusetts Medical School, Worcester, MA USA

**Keywords:** Childhood obesity, Socioeconomic status, Home environment, Sleep, Socioecologic model

## Abstract

**Background:**

Childhood obesity disproportionally affects children from low-income households. With the aim of informing interventions, this study examined pathways through which the physical and social home environment may promote childhood overweight/obesity in low-income households.

**Methods:**

Data on health behaviors and the home environment were collected at home visits in low-income, urban households with either only normal weight (n = 48) or predominantly overweight/obese (n = 55) children aged 6–13 years. Research staff conducted comprehensive, in-person audits of the foods, media, and sports equipment in each household. Anthropometric measurements were collected, and children’s physical activity was assessed through accelerometry. Caregivers and children jointly reported on child sleep duration, screen time, and dietary intake of foods previously implicated in childhood obesity risk. Path analysis was used to test direct and indirect associations between the home environment and child weight status via the health behaviors assessed.

**Results:**

Sleep duration was the only health behavior associated with child weight status (OR = 0.45, 95% CI: 0.27, 0.77), with normal weight children sleeping 33.3 minutes/day longer on average than overweight/obese children. The best-fitting path model explained 26% of variance in child weight status, and included paths linking chaos in the home environment, lower caregiver screen time monitoring, inconsistent implementation of bedtime routines, and the presence of a television in children’s bedrooms to childhood overweight/obesity through effects on screen time and sleep duration.

**Conclusions:**

This study adds to the existing literature by identifying aspects of the home environment that influence childhood weight status via indirect effects on screen time and sleep duration in children from low-income households. Pediatric weight management interventions for low-income households may be improved by targeting aspects of the physical and social home environment associated with sleep.

## Background

Childhood obesity disproportionally affects low-income children [1], which may contribute to socioeconomic disparities in obesity-related chronic diseases throughout the lifespan [2]. Socioecologic models attribute childhood obesity to intersecting social, economic, environmental, and psychobiologic drivers of energy intake and expenditure [3]. Prior work based on this model has largely focused on neighborhood-level factors, such as local access to healthy and unhealthy foods, geographic density of fast food outlets and supermarkets, and venues for physical activity in relation to childhood obesity risk [4, 5]. A few recent studies have highlighted the role of the *home environment* in childhood obesity, including both its physical features and social processes involving children and caregivers [6]. Physical home environments characterized by greater availability of unhealthy foods, fewer fruits and vegetables, more media equipment throughout the home and in the child’s bedroom, and fewer sports/recreational equipment items have been linked to childhood obesity risk [7–10]. Aspects of the social home environment, including caregiver modeling and policies towards healthy eating and physical activity, are also important influences [11, 12].

Very little research to date has examined childhood obesity risk factors in the home environments of low-income households. In particular, little is known about aspects of the home environment that are associated with short sleep duration, which is highly prevalent among low-income minority youth [13] and has been consistently associated with weight gain and obesity status in prospective and cross-sectional studies [14–16]. This study compared the home environments of normal weight and overweight/obese children from low-income households. Consistent with socioecologic models of health and prior studies in non-disadvantaged populations, it was hypothesized that features of the physical and social home environment associated with healthy dietary intake, increased physical activity, reduced screen time, and longer sleep duration would discriminate between low-income households with exclusively normal weight children from those with predominantly overweight/obese children. An improved understanding of the features of the home environment most strongly associated with childhood obesity in low-income households could be leveraged to develop novel pediatric obesity interventions for this population. By comparing normal weight versus overweight/obese children within an entirely low-income population, this study reduced confounding by household income, and ensured that any therapeutic changes to the home environment suggested by the findings would be financially feasible for low-income families.

## Methods

This manuscript reports the primary analyses from the *Home Environment Comparison Study,* a cross-sectional investigation of home environmental childhood obesity risk factors in low-income, urban households. Data were collected in Chicago, IL, USA during 2012–2013.

### Subjects

Households were recruited through posted advertisements, pediatrician referrals, and word-of-mouth between May 2012 and March 2013. Eligible households were located in the city of Chicago, had at least one child between ages 6 and 13 years, reported a household income ≤250% of the Federal Poverty Threshold (FPT; <$57,625/year for a 4-member household), and included an adult caregiver who made the majority of household food purchases and was willing to participate (index caregiver). Households also met criteria as cases or controls. In overweight/obese households (cases), at least 50% of children had a body mass index ≥85th percentile for their age and sex (consistent with the Centers for Disease Control and Prevention’s definition [17]). In normal weight households (controls), all children ages 6 to 18 years had a body mass index <85th percentile for their age and sex. Households in which 1%-49% of children ages 6 to 18 were overweight or obese were excluded to maximize observed group differences in home environments. Data collection focused on children ages 6–13 due to expectation that the diet, activity levels, and sleep patterns of children ages 14–18 may be less influenced by the physical and social home environment than younger children. To maximize observed group differences and reduce respondent burden in multiple-child households, data collection focused on one index child per household who had either the highest (overweight/obese weight households) or lowest (normal weight households) BMI percentile among children ages 6–13.

Exclusion criteria were selected to eliminate potential confounds and reduce barriers to data collection, including: 1) serious physical illness or developmental problem in any child age 6 to 13 (e.g., paraplegia, autism), 2) serious physical or psychiatric illness in a primary caregiver, 3) living in temporary or group housing or planning to move within 2 months, 4) lack of reliable telephone access, 5) lack of verbal English fluency, 6) and unwilling to meet with researchers in the home. Of 154 households who inquired about the study, 4 (3%) declined screening, 22 (14%) were eligible but did not enroll due to scheduling difficulties, and 25 (16%) were excluded based on child weight status (n = 11), income >250% of FPT (n = 8), living outside Chicago (n = 4), living in temporary housing (n = 1), or because the primary household food shopper was unavailable to participate (n = 1). The final sample included 103 households.

The study was conducted in accordance with the Declaration of Helsinki, and Rush University Medical Center’s Institutional Review Board approved study procedures. Researchers obtained written documentation of informed consent and child assent. Caregivers were compensated $60.00.

### Measures

#### Anthropometric measurements

Index caregiver and index child height and weight were measured in light clothing without shoes using a scale and stadiometer (SECA models 876 and 213, Hamburg, Germany). Body mass index (BMI; kg/m^2^) was calculated, and for children, BMI percentile for age and sex was determined.

#### Physical activity

The index child’s engagement in moderate and vigorous physical activity was assessed through 7-day triaxial accelerometry (ActiGraph GT3X+; Actigraph LLC, Pensacola, FL). Periods of no activity ≥20 minutes were deemed non-wear times. To be included in analyses, subjects had to have at least 10 hours of valid wear time per day on a minimum of 3 weekdays and 1 weekend day [18]. Minutes of moderate and vigorous physical activity were calculated for each complete day of data using validated scoring criteria [19]. The weighted average of weekday (weight = 5/7 or 0.714) and weekend (weight = 2/7 or 0.286) physical activity was calculated.

#### Screen time

Items adapted from the Center for Disease Control and Prevention’s 2011 State and Local Youth Risk Behavior Survey [20] were adapted to assess time spent watching television or movies, and playing video games or engaging in recreational computer use on typical school days and weekends. For ease of interpretation, analyses utilized the weighted average of weekday and weekend screen time based on the midpoints of each response category (0, 0.5, 1.5, 2.5, and 3.5 hours per day).

#### Index child dietary intake

Caregivers and index children jointly reported on child consumption of specific categories of foods linked to obesity risk [21–23] during the past week on a 9-point scale from “Never” to “5 or more times per day”. Frequency of fruit and vegetable intake was assessed using four items and the response format from the National Cancer Institute’s Fruit and Vegetable Screener [24], which included 100% fruit juice, fruit, lettuce salad, and vegetables other than lettuce salad or fried potatoes. Intake of discretionary caloric beverages (regular soda, sports drinks, other sweetened beverages) and fast food during the past week were assessed with a subset of items from a validated screener [25]. Intake of seven categories of energy-dense snacks and desserts (chips/salty snack foods; candy; ice cream/frozen treats; cookies; cake/cupcakes; brownies/dessert bars/muffins; donuts/pastries) from a validated home food inventory [26] (described below) were assessed using the same response format. Total scores for fruit and vegetable intake, fast food and discretionary caloric beverage intake, and energy-dense snack intake were calculated.

#### Sleep duration

The Sleep Habits Survey [27] was used to assess usual total sleep time over the past two weeks. The Sleep Habits Survey has been validated against sleep actigraphy and sleep diaries in children [28]. Responses were provided by the index child with the assistance of caregivers, as caregivers tend to overestimate child sleep duration [29]. Usual sleep duration was calculated as the weighted average of weekday and weekend sleep durations in hours per day (h/d).

#### Household characteristics and socioeconomic status

Household income, household size, index caregiver education level, and child and caregiver gender and race/ethnicity were assessed via self-report. Household income was quantified as a percentage of the Federal Poverty Threshold (FPT), which considers income relative to household size and composition.

#### Home food environment

A comprehensive, validated home food environment auditing tool was completed by research staff [26]. The tool assesses the presence and accessibility of 190 food items in 13 food group categories, including fruits (20 items), vegetables (26 items), and 71 calorie-dense indicator foods that contribute to an overall “obesogenic food availability score”.

#### Home activity and media environment

Research staff completed a room-by-room audit using the Physical Activity and Media Inventory [9, 30], which quantified the total number of media (5 items) and sports/recreational equipment (50 items) throughout the home. The presence of a television in the index child’s bedroom was also recorded [10].

#### Social home environment

Items from the Family Nutrition & Physical Activity Screening Tool assessed the frequency of family practices associated with childhood obesity [31]. The measure has 20 items scored from “Almost Never” (1) to “Almost Always” (4). Given our interest in specific caregiver behaviors, final path models utilized items and subscale scores for caregiver screen time monitoring (3 items; Cronbach α = 0.67) and consistent implementation of a bedtime routine [“almost always” (n = 58) versus “almost never”, “sometimes”, or “usually” (n = 45)].

#### Chaotic home environment

Index caregiver’s perception of chaos and disorganization in the home environment was assessed using the Confusion, Hubbub, and Order Scale [32]. This measure has 15 true/false items that are summed to obtain total score (Cronbach α = 0.81).

### Data analysis

Variable distributions were evaluated for normality and extreme values using skew and kurtosis indexes and normal quantile plots. Preliminary confirmatory factor analyses indicated that the three dietary intake variables (fruits and vegetables, discretionary caloric beverages and fast food, and energy-dense snacks) could not be reduced to one or two latent variables representing dietary intake. They were therefore treated as separate, observed variables. Logistic regression models tested associations between selected home environment variables and child weight status.

Our primary analyses were conducted in two stages. First, we sought to determine which proximal health behaviors were associated with child weight status in low-income households. Associations between child weight status and six measured health behaviors (moderate and vigorous physical activity, screen time, sleep duration, and dietary intake of fruits and vegetables, discretionary caloric beverages and fast food, and energy-dense snacks) tested separately in logistic regression models with and without adjustment for index caregiver BMI.

In the second stage of analysis, path analysis was used to develop and test a theory-driven model linking aspects of the physical and social home environment to child weight status via effects on health behaviors associated with child weight status in the preceding analyses. Paths were added or removed from the model based on both theoretical considerations and model fit indices. Model chi-square with a p > .05, comparative fit index (CFI) ≥ .90, and root mean square error of approximation (RMSEA) ≤0.05 indicated good model fit [33]. Missing data were handled using full-information maximum likelihood.

Given the strong heritability of adiposity [34, 35] and the possibility that caregivers may transmit risk for overweight/obesity to their children through effects on the home environment, effects of index caregiver BMI on child weight status were directly modeled in both stages of analysis. Child age, household income, and caregiver education were also considered as covariates. Mean- and variance-adjusted weighted least squares estimation was used because the dependent variable of child weight status was dichotomous, and this estimation technique is robust to non-normality in dependent variables [33]. Unstandardized path coefficients (b), standard errors, and p-values are reported, along with standardized path coefficients (β) to facilitate interpretation of associations. Descriptive analyses and logistic regression models were conducted with Stata 11 (StataCorp LP, College Station, TX). Structural modeling was conducted with Mplus 7.11 (Muthén & Muthén, Los Angeles, CA).

## Results

The sample included 103 enrolled households (Table 1), 48 (47%) of which were normal weight households and 55 (53%) of which were overweight/obese households. Accelerometer data from 88 index children (85% of total; 81% of normal weight households, 89% of overweight households) met *a priori* criteria for valid wear time and were included in analyses. Complete data (N = 103) were available for all other variables. No variable distribution had a skew index >2.0 or a kurtosis index >7.0, which are conservative guidelines for problematic non-normality in structural equation modeling [33]. Means, standard deviations, and zero-order correlations among study variables included in structural models are reported in Table 2.

**Table 1:** **Characteristics of enrolled households**

	Total (N = 103)	Normal weight (n = 48)	Overweight (n = 55)	***p***-value ^a^
**Household**				
Household size [M (SD)]	3.9 (1.7)	3.9 (1.8)	4.0 (1.6)	.80
Income, % FPT [M (SD)]^b^	107.0 (76.6)	99.3 (79.4)	113.7 (74.2)	.35
**Index child**				
Age, years [M (SD)]	10.0 (2.5)	9.8 (2.5)	10.1 (2.5)	.58
BMI percentile [M (SD)]	73.6 (29.6)	48.2 (25.5)	95.8 (3.9)	<.0001
Female gender [n (%)]	54 (52)	23 (48)	31 (56)	.39
Race/ethnicity [n (%)]				.88
Black/African-American	79 (77)	37 (77)	42 (76)	
Hispanic/Latino	18 (18)	9 (19)	9 (16)	
Multi-ethnic/Other	3 (3)	1 (2)	2 (4)	
Non-Hispanic White/Caucasian	2 (2)	1 (2)	1 (2)	
Asian	1 (1)	0 (0)	1 (2)	
Receives subsidized school meals	94 (91.3)	43 (89.6)	51 (92.7)	.32
Fruit & vegetables (range: 0–32)	11.9 (4.1)	11.9 (4.3)	11.9 (3.9)	.91
Fast food & caloric beverages (range: 0–32)	9.2 (3.5)	8.8 (3.4)	9.5 (3.5)	.30
Energy-dense snacks (range: 0–56)	15.6 (6.0)	15.5 (5.3)	15.8 (6.5)	.80
MVPA (mins/d; n = 88)	48.4 (22.2)	49.2 (18.6)	47.7 (24.9)	.76
Screen time (h/d)	3.5 (1.5)	3.3 (1.4)	3.7 (1.5)	.13
Sleep duration (h/d)	9.5 (0.9)	9.7 (0.9)	9.2 (0.9)	<.01
Caregiver screen time monitoring (range: 3–12)	7.7 (2.1)	8.2 (2.0)	7.2 (2.1)	.01
Chaotic home environment (range: 0–15)	3.2 (3.1)	2.7 (2.9)	3.6 (3.3)	.13
**Index caregiver**				
Age, years [M (SD)]	36.5 (7.4)	36.2 (7.2)	36.7 (7.6)	0.72
BMI, kg/m^2^ [M (SD)]	33.5 (9.6)	30.5 (9.3)	36.0 (9.3)	<.01
Female gender [n (%)]	97 (94)	44 (92)	53 (96)	.31
Race/ethnicity [n (%)]				0.83
Black/African-American	79 (77)	37 (77)	42 (76)	
Hispanic/Latino	17 (17)	8 (17)	9 (16.4)	
Multi-ethnic/Other	1 (1)	0 (0)	1 (2)	
Non-Hispanic White/Caucasian	6 (6)	3 (6)	3 (6)	
Asian	0 (0)	0 (0)	0 (0)	
Education [n (%)]				.14
High school or lower	25 (24)	15 (31)	10 (18)	
Some college/technical school	64 (62)	25 (52)	39 (71)	
4-year degree or higher	14 (14)	8 (17)	6 (11)	

FPT, Federal Poverty Threshold; BMI, body mass index; MVPA, moderate and vigorous physical activity.

^a^For tests of group differences through t-test or chi-square test.

^b^FPTs are set by the U.S. Census Bureau based on annual household income and the number of related adults and children in the household.

**Table 2:** **Correlations among continuous variables included in path analyses (N = 103 unless otherwise noted)**

	Pearson correlations						
	2	3	4	5	6	7	8
1. Fruit & vegetables	.14	.28**	.05	-.09	.13	.12	-.03
2. Fast food & caloric beverages	--	.46***	.08	.33***	-.17	-.21*	.07
3. Energy-dense snacks		--	.21	.26**	-.15	.15	-.08
4. MVPA^a^			--	-.13	.16	.23*	-.16
5. Screen time				--	-.33***	-.37***	.07
6. Sleep duration					--	.13	-.23*
7. Caregiver screen time monitoring						--	-.17
8. Chaotic home environment							--

*p < .05, **p < .01, ***p < .001.

MVPA, daily minutes of moderate and vigorous physical activity.

^a^ N=88 for this variable.

In the first stage of our analyses (Table 3), sleep duration was the only measured health behavior that was significantly associated with child weight status (unadjusted OR = 0.45, 95% CI: 0.27, 0.77, p = <.01). On average, normal weight children (9.7 h/d) slept 33.3 minutes longer per night than overweight/obese index children (9.2 h/d). The association was unchanged with adjustment for index caregiver BMI (adjusted OR = 0.45, 95% CI: 0.26, 0.77, p = <.01). Higher index caregiver BMI was associated with greater odds of child overweight/obese status (adjusted OR = 1.07, 95% CI: 1.02, 1.13, p = <.01).

**Table 3:** **Associations between health behaviors and odds of child overweight/obese weight status, with and without adjustment for index caregiver BMI (N = 103)**

	Unadjusted			Adjusted for caregiver BMI		
	OR	95% CI	***p***-value	OR	95% CI	***p***-value
Fruit and vegetable intake^a,b^	1.01	0.91, 1.11	.91	1.03	0.93, 1.14	.59
Caloric beverages and fast food^a,b^	1.06	0.95, 1.20	.30	1.07	0.95, 1.21	.27
Energy-dense snacks^a,c^	1.01	0.94, 1.08	.80	1.02	0.95, 1.09	.55
Screen time (h/d)	1.23	0.94, 1.61	.14	1.17	0.89, 1.57	.26
MVPA (mins/d)^d^	1.00	0.98, 1.02	.76	1.00	0.98, 1.02	.92
Sleep duration (h/d)	0.45	0.27, 0.77	<.01	0.45	0.26, 0.77	<.01

MVPA, daily minutes of moderate and vigorous physical activity.

^a^Measured on an ordinal scale reflecting frequency of intake in the past 7 days.

^b^Possible scores ranged from 0–32.

^c^Possible scores ranged from 0–56.

^d^N = 88 for this variable.

**Figure 1:**
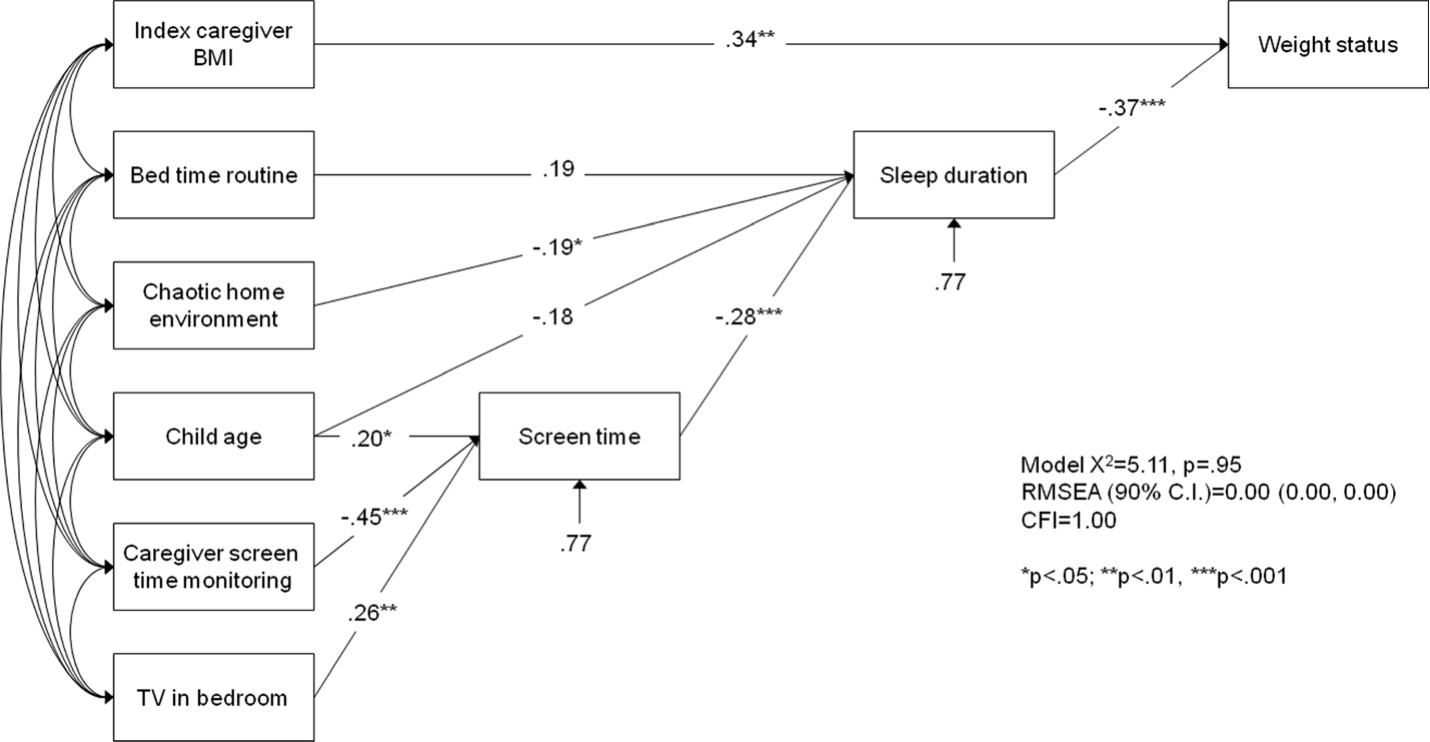
**Final path model linking features of the home environment to childhood overweight/obese status through sleep duration (N = 103).** Values are standardized path coefficients.

The second stage of analysis focused on modeling associations between the social and physical home environment and child weight status through mediating paths involving sleep duration. Variables included in the initial model were total media items in the home, the presence of a television in the index child’s bedroom, screen time (as an influence on sleep duration), caregiver screen time monitoring, consistent implementation of a bedtime routine, perceived chaos and disorganization in the home environment, and several covariates (index caregiver BMI, child age, household income, index caregiver education). Total media items in the home was not associated with sleep duration or screen time and was removed from the model. The final path model (Table 4; Figure 1; model χ^2^ = 5.11, p = .95; CFI = 1.00; RMSEA = 0.00) explained 26% of variance in weight status, and 23% of the variance in both sleep duration and screen time.

**Table 4:** **Unstandardized (b) and standardized (β) path coefficients for final model** ^**a**^ **of home environment variables, sleep duration, and child overweight/obese status (N = 103)**

	b	SE	β	***p***-value
Direct effects on weight status				
Index caregiver BMI	.04	.01	.34	<.01
Sleep duration	-.44	.11	-.37	<.001
Direct effects on sleep duration				
Index child age	-.07	.04	-.18	.08
CHAOS	-.06	.03	-.19	.03
Bedtime routine	.34	.20	-.19	.08
Screen time	-.17	.05	-.28	<.001
Direct effect on screen time				
TV in bedroom	.85	.33	.26	<.01
CSM	-.31	.08	-.45	<.001
Index child age	.12	.06	.20	.04
Indirect effects				
CHAOS → Sleep → Weight status	.03	.01	.07	.06
Bedtime routine → Sleep → Weight status	-.15	.09	-.07	.10
Screen time → Sleep → Weight status	.08	.03	.10	<.01
CSM → Screen time → Sleep → Weight status	-.02	.01	-.05	.02
TV → Screen time → Sleep → Weight status	.07	.03	.03	.06

BMI, body mass index.

CSM, caregiver screen time monitoring.

CHAOS, chaos and disorganization in the home environment.

TV, TV present in index child’s bedroom.

^a^Model χ^2^ = 5.11, p = .95; CFI = 1.00; RMSEA = 0.00, 90% CI: 0.00, 0.00.

Finally, we examined associations between child weight status and several home environment variables that were not evaluated or retained in the stage two analyses. Home availability of fruits (OR = 0.97, 95% CI: 0.87, 1.09, p = .62), vegetables (OR = 0.93, 95% CI: 0.80, 1.08, p = .35), and obesity-promoting foods (OR = 1.02, 95% CI: 0.96, 1.08, p = .52) were not associated with child weight status. Similarly, the home availability of sports/recreational (OR = 0.96, 95% CI: 0.89, 1.03, p = .25) and media (OR = 1.01, 95% CI: 0.88, 1.15, p = .91) equipment were not associated with child weight status.

## Discussion

This is the first study to examine how objectively-measured features of the physical home environment and aspects of the social home environment are related to child weight status through their influences on health behaviors in an entirely low-income population. Specifically, our findings highlight the potential importance of targeting sleep in weight management interventions for low-income children by promoting consistent implementation of a bedtime routine, reducing chaos and disorganization in the home environment, and encouraging caregiver monitoring of screen time. Our data also support removing televisions from children’s bedrooms, which is commonly addressed in pediatric weight management interventions. In contrast to prior studies [9], the availability of sports/recreational equipment in the home was not associated with weight status. If this result reflects a true lack of association in the population, rather than insufficient statistical power, it would suggest that increasing access to sports/recreational equipment is unlikely to promote weight loss in low-income children, even though these items are less abundant in low-income households than higher-income households [36].

Several associations observed in our sample of low-income children converge with prior studies in different populations. A recent Australian study reported that a longitudinal association between short sleep duration at ages 4–5 years and higher adiposity at ages 8–9 years was partially mediated by increases in television viewing time in the intervening years [37]. Another study involving third through fifth graders in Iowa and Minnesota, USA found that parental monitoring of media use was associated with longer sleep duration through indirect effects on screen time [38]. The current findings indicate that sleep duration and screen time are important influences on weight status in children from low-income households (and are not simply artifacts of confounding by socioeconomic status), and also provides new information on the features of the home environment that may influence these behaviors in this population.

Very few published pediatric weight management interventions include a strong focus on modifying the home environment. One exception is a recent trial involving predominantly low-income children ages 2–5 years, which reported that a 6-month, home-based, health education intervention focused on adopting healthy family routines led to longer sleep duration, decreased television viewing, and a small but positive effect on BMI [39]. Our group is currently pilot testing a home-based, parent-only weight management intervention for low-income children ages 6–13 years that targets children’s sleep, dietary intake, and physical activity through guided modification of the home environment and parent skills training.

Traditional childhood obesity risk factors such as physical activity, screen time, and intake of discretionary caloric beverages and fast food, energy-dense snacks, and fruits and vegetables, were not directly related to weight status in this study. Reduced variability in these behaviors may account for the lack of observed associations, as low-income children’s physical activity and diet may be constrained by school physical education policies, reliance on subsidized school meals (91% of index children received free/reduced school meals), local access to healthy food and physical activity venues, and socioeconomic influences on household food choices. The presence of such constraints may result in sleep accounting for a larger proportion of variance in weight status in this population. Disparities in childhood obesity have been attributed to differences in diet, screen time, and physical activity in studies that compare high- and low-income subjects, but these factors have been less consistently associated with weight status in other studies that include only low-income subjects [40]. The current findings suggest that the health behaviors that underlie socioeconomic disparities in childhood obesity differ from those associated with obesity within an entirely low-income population.

A major strength of this study was the use of objective audit-based measures of the physical home environment, which eliminates the reporting bias that can occur with self-report measures. The use of in-home data collection also eliminated lack of transportation or childcare as barriers to participation, which is important in low-income populations. The study also examined childhood obesity risk factors exclusively among low-income households eliminates income as a confounding factor and enables the development of home-based interventions that are compatible with the resources of low-income households.

This study also had several limitations. The sample was composed primarily of African-American households in Chicago, and a different pattern of findings may be observed in other populations. The use of a convenience sampling methods (e.g., advertisements, pediatrician referrals) further limits generalizability to the broader population. Our eligibility criteria focused on household income as the sole determinant of socioeconomic position. Though our sample was 98% ethnic minority and caregivers had a low-level of educational attainment, confounding by other aspects of socioeconomic position such as occupational class, wealth, and acculturation were not addressed. The sample size was small, which limited statistical power, precluded moderator analyses, and reduced the stability of path coefficients. As a result, non-significant associations should not be interpreted as a definitive indication that no such association exists in the population. Sleep duration was assessed with a validated self-report measure of bedtimes and waketimes, which tends to overestimate sleep duration relative to sleep actigraphy [28]. We also did not assess sleep disordered breathing, which could represent a reverse causal pathway in which weight status leads to short sleep duration. The lack of observed associations with dietary intake may stem from our focus on the frequency of intake of specific food categories. Our measures did not capture portion size, overall energy intake, or diet quality. The cross-sectional nature of the study cannot assess causality, and prospective studies examining the roles of the home environment, screen time, and sleep on obesity in low-income children are warranted. Finally, an inherent limitation of path analysis is that it can only compare alternative theoretical models based on their relative fit with observed data, and decisions about which models to test require some subjective judgment on the part of the investigators [33].

## Conclusion

The main finding from this study is that several aspects of the physical and social home environment were related to childhood weight status via their indirect associations involving sleep duration and screen time. Interventions that explicitly target these features of the home environment could augment pediatric weight management interventions in low-income populations.

## Abbreviations

BMI: Body mass index
CFI: Comparative fit index
CI: Confidence interval
FPT: Federal poverty threshold
OR: Odds ratio
RMSEA: Root mean square error of approximation.
